# Association between primary or booster COVID-19 mRNA vaccination and Omicron lineage BA.1 SARS-CoV-2 infection in people with a prior SARS-CoV-2 infection: A test-negative case–control analysis

**DOI:** 10.1371/journal.pmed.1004136

**Published:** 2022-12-01

**Authors:** Margaret L. Lind, Alexander J. Robertson, Julio Silva, Frederick Warner, Andreas C. Coppi, Nathan Price, Chelsea Duckwall, Peri Sosensky, Erendira C. Di Giuseppe, Ryan Borg, Mariam O. Fofana, Otavio T. Ranzani, Natalie E. Dean, Jason R. Andrews, Julio Croda, Akiko Iwasaki, Derek A. T. Cummings, Albert I. Ko, Matt D. T. Hitchings, Wade L. Schulz

**Affiliations:** 1 Department of Epidemiology of Microbial Diseases, Yale School of Public Health, New Haven, Connecticut, United States of America; 2 Department of Immunobiology, Yale University School of Medicine, New Haven, Connecticut, United States of America; 3 Section of Cardiovascular Medicine, Department of Internal Medicine, Yale School of Medicine, New Haven, Connecticut, United States of America; 4 Center for Outcomes Research and Evaluation, Yale-New Haven Hospital, New Haven, Connecticut, United States of America; 5 Barcelona Institute for Global Health, ISGlobal, Universitat Pompeu Fabra (UPF), Barcelona, Spain; 6 CIBER Epidemiología y Salud Pública (CIBERESP), Madrid, Spain; 7 Pulmonary Division, Heart Institute, Hospital das Clínicas, Faculdade de Medicina, São Paulo, Brazil; 8 Department of Biostatistics & Bioinformatics, Rollins School of Public Health, Emory University, Atlanta, Georgia, United States of America; 9 Division of Infectious Diseases and Geographic Medicine, Stanford University, Stanford, California, United States of America; 10 Fiocruz Mato Grosso do Sul, Fundação Oswaldo Cruz, Campo Grande, Brazil; 11 Universidade Federal de Mato Grosso do Sul, Campo Grande, Brazil; 12 Howard Hughes Medical Institute, Chevy Chase, Maryland, United States of America; 13 Department of Biology, University of Florida, Gainesville, Florida, United States of America; 14 Emerging Pathogens Institute, University of Florida, Gainesville, Florida, United States of America; 15 Instituto Gonçalo Moniz, Fundação Oswaldo Cruz, Salvador, Brazil; 16 Department of Biostatistics, College of Public Health and Health Professions & College of Medicine, University of Florida, Gainesville, Florida, United States of America; 17 Department of Internal Medicine, Yale School of Medicine, New Haven, Connecticut, United States of America; 18 Department of Laboratory Medicine, Yale University School of Medicine, New Haven, Connecticut, United States of America; Washington University in St Louis School of Medicine, UNITED STATES

## Abstract

**Background:**

The benefit of primary and booster vaccination in people who experienced a prior Severe Acute Respiratory Syndrome Coronavirus 2 (SARS-CoV-2) infection remains unclear. The objective of this study was to estimate the effectiveness of primary (two-dose series) and booster (third dose) mRNA vaccination against Omicron (lineage BA.1) infection among people with a prior documented infection.

**Methods and findings:**

We conducted a test-negative case–control study of reverse transcription PCRs (RT-PCRs) analyzed with the TaqPath (Thermo Fisher Scientific) assay and recorded in the Yale New Haven Health system from November 1, 2021, to April 30, 2022. Overall, 11,307 cases (positive TaqPath analyzed RT-PCRs with S-gene target failure [SGTF]) and 130,041 controls (negative TaqPath analyzed RT-PCRs) were included (median age: cases: 35 years, controls: 39 years). Among cases and controls, 5.9% and 8.1% had a documented prior infection (positive SARS-CoV-2 test record ≥90 days prior to the included test), respectively. We estimated the effectiveness of primary and booster vaccination relative to SGTF-defined Omicron (lineage BA.1) variant infection using a logistic regression adjusted for date of test, age, sex, race/ethnicity, insurance, comorbidities, social venerability index, municipality, and healthcare utilization. The effectiveness of primary vaccination 14 to 149 days after the second dose was 41.0% (95% confidence interval (CI): 14.1% to 59.4%, *p* 0.006) and 27.1% (95% CI: 18.7% to 34.6%, *p* < 0.001) for people with and without a documented prior infection, respectively. The effectiveness of booster vaccination (**≥**14 days after booster dose) was 47.1% (95% CI: 22.4% to 63.9%, *p* 0.001) and 54.1% (95% CI: 49.2% to 58.4%, *p* < 0.001) in people with and without a documented prior infection, respectively. To test whether booster vaccination reduced the risk of infection beyond that of the primary series, we compared the odds of infection among boosted (**≥**14 days after booster dose) and booster-eligible people (**≥**150 days after second dose). The odds ratio (OR) comparing boosted and booster-eligible people with a documented prior infection was 0.79 (95% CI: 0.54 to 1.16, *p* 0.222), whereas the OR comparing boosted and booster-eligible people without a documented prior infection was 0.54 (95% CI: 0.49 to 0.59, *p* < 0.001). This study’s limitations include the risk of residual confounding, the use of data from a single system, and the reliance on TaqPath analyzed RT-PCR results.

**Conclusions:**

In this study, we observed that primary vaccination provided significant but limited protection against Omicron (lineage BA.1) infection among people with and without a documented prior infection. While booster vaccination was associated with additional protection against Omicron BA.1 infection in people without a documented prior infection, it was not found to be associated with additional protection among people with a documented prior infection. These findings support primary vaccination in people regardless of documented prior infection status but suggest that infection history may impact the relative benefit of booster doses.

## Abstract 2

Using a Test-Negative Case-Control Analysis, Dr Margaret L. Lind and colleagues, investigate the association between primary or booster COVID-19 mRNA vaccination and omicron lineage BA.1 SARS-CoV-2 infection in people with a prior SARS-CoV-2 infection.

## Author summary

**Why was this study done?:**

- mRNA COVID-19 vaccines provide lower levels of protection against Omicron (BA.1 lineage) infections than previously circulating variants.
- Prior to the emergence of the Omicron variant, primary vaccination (first two mRNA vaccine doses) afforded protection against reinfection among people with prior infections.
- The benefit of primary and booster vaccination against Omicron (BA.1 lineage) infections remains unclear among people with prior infections.

**What did the researchers do and find?:**

- We evaluated the benefit of primary series and booster mRNA vaccine doses against Omicron (BA.1 lineage, defined by S-gene target failure) infection among people with and without documented prior infections.
- We found that primary vaccination was associated with statistically significant but low levels of protection against Omicron (BA.1 lineage) infection among people with and without a documented prior infection.
- Booster vaccination was found to be associated with protection beyond that afforded by the primary series among people without a documented prior infection, but we did not observe a significant increase in protection among people with a documented prior infection.

**What do these findings mean?:**

- Primary vaccination provides limited but significant protection against Omicron (BA.1 lineage) infection regardless of prior infection history.
- The relative benefits of a booster dose may be affected by a person’s history of prior SARS-CoV-2 infection.

## Introduction

Although Coronavirus Disease 2019 (COVID-19) vaccines provide lower levels of protection against the B.1.1.529 (Omicron) than the B.1.617.2 (Delta) variant of Severe Acute Respiratory Syndrome Coronavirus 2 (SARS-CoV-2), current evidence indicates that primary and booster mRNA (third) vaccination significantly reduces the risk of Omicron-related infection and poor outcomes in the general population [1–6]. However, the benefit of vaccination in people with a prior SARS-CoV-2 infection remains uncertain. Previous studies, conducted prior to the Omicron epidemic wave, found that primary vaccination (two doses) afforded protection against reinfection beyond that provided by a prior infection [7–10] and that a booster dose significantly increase such protection [11]. In contrast, Shrestha and colleagues found that primary vaccination did not provide additional protection (hazard ratio, 0.77, 95% confidence interval (CI): 0.53 to 1.12) against SARS-CoV-2 reinfection among previously infected people during the first month of the Omicron wave [12]. Furthermore, evidence is lacking for the additional benefit of booster vaccination against Omicron infection in people with a documented prior infection, which is needed to inform vaccination policies for this subpopulation.

In this study, we analyzed data from a large cohort of people receiving care in the Yale New Haven Health system who underwent molecular testing for S-gene target failure (SGTF) to evaluate the benefit of primary series and booster doses in the context of the Omicron wave. Specifically, we estimated the effectiveness of primary and booster vaccination against Omicron (lineage BA.1) infection among people with and without a documented prior SARS-CoV-2 infection. We also examined whether booster vaccination reduced the risk of Omicron (lineage BA.1) infection beyond that afforded by primary vaccination among people with and without a prior documented infection.

## Methods

### Study setting and population

We conducted a test-negative case–control (TNCC) analysis using data collected as part of the Studying COVID-19 Outcomes after SARS-CoV-2 Infection and Vaccination (SUCCESS) Study in the Yale New Haven Health System (YNHH). The study was designed in January 2022 and executed in May 2022. As part of the peer review process, Wald test *P* values were extracted from the described regressions, the unadjusted analyses were performed, and the bias indicator was added. The YNHH is a large academic health system comprising five hospital delivery networks and associated outpatient clinics in Connecticut, New York, and Rhode Island. We chose the TNCC design because it has been shown to provide effectiveness estimates consistent with those from randomized controlled trials, has been widely applied to estimate real-world effectiveness for COVID-19 vaccines, and mitigates the risk of confounding introduced by differences in care-seeking and testing access [1,13–16].

**Fig 1:**
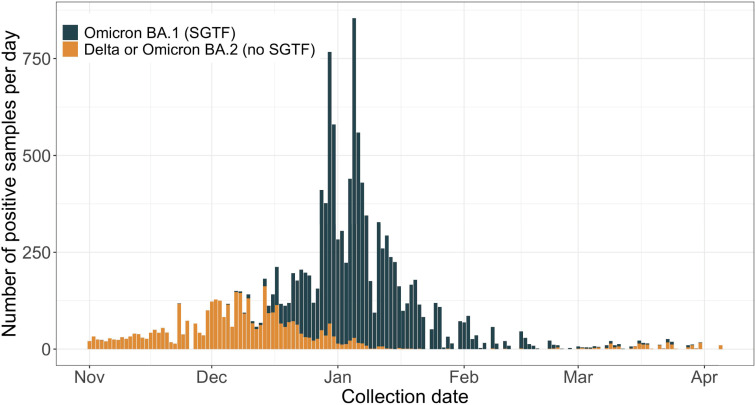
Daily Omicron lineage BA.1 (SGTF) and other variant (non-SGTF) SARS-CoV-2 infections identified during TaqPath testing at the YNHH between November 1, 2021 and April 30, 2022. TaqPath COVID-19 (Thermo Fisher Scientific) confirmed SARS-CoV-2 infections among vaccine-eligible individuals. Infections were classified as Omicron (lineage BA.1) based on presence of SGTF. COVID-19, Coronavirus Disease 2019; SARS-CoV-2, Severe Acute Respiratory Syndrome Coronavirus 2; SGTF, S-gene target failure; YNHH, Yale New Haven Health System.

The study population comprised vaccine-eligible (**≥**5 years of age) people who were alive at the beginning of the study period and had at least one SARS-CoV-2 test in the electronic medical records (EMRs). We identified SARS-CoV-2 reverse transcription PCR (RT-PCR) tests that were collected from the study population and performed with the TaqPath COVID-19 (Thermo Fisher Scientific) diagnostic assay between November 1, 2021 and April 30, 2022, the period prior to and during the Omicron (lineage BA.1) epidemic wave in Connecticut (Fig 1). At the beginning of the study, Delta was the predominant variant in Connecticut, accounting for 99.63% (3,808 of 3,822) of the sequenced samples deposited in the GISAID database that were collected between November 1 and November 28, 2021 [17]. We used the TaqPath assay to select tests as cases and controls since its S-gene probe, which fails for Omicron (lineage BA.1) but not for Delta, allows for prediction of an Omicron (lineage BA.1) infection when the primary circulating variants are Omicron (lineage BA.1) and Delta [18].

**Fig 2:**
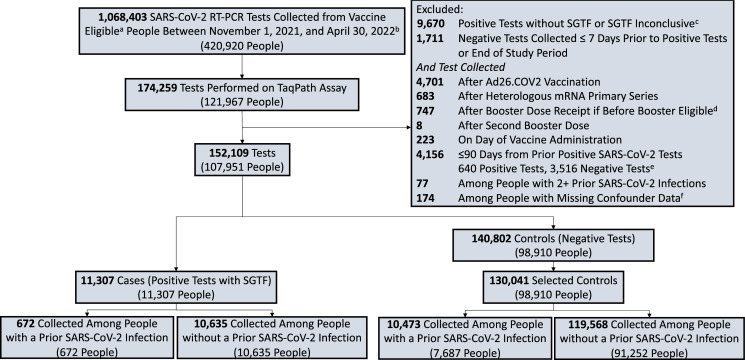
Selection of tests for the case–control analysis. The sample was limited to RT-PCRs run on the TaqPath COVID-19 (Thermo Fisher Scientific) assay among vaccine-eligible individuals. Case status was defined based on the reflex results. We included all positive tests (cases) and up to three negative tests (controls) per person. Cases and controls were stratified by presence of a documented prior infection (a positive RT-PCR or rapid antigen test at least 90 days before the included test). ^a^Vaccine eligibility was defined as age ≥5 years. ^b^The first SGTF defined Omicron (BA.1 lineage) variant infection in the study population was identified on November 11, 2021. ^c^Samples with a positive reflex RT-PCR but that did not meet our SGTF definition (an ORF1ab Ct value of <30 and S-gene Ct–ORF1ab Ct value ≥5; or 2] ORF1ab Ct value <30 and S-gene Ct value ≥40. ^d^Excluded tests that were performed after a person was given a booster dose before FDA authorization or that was given less than 150 days after primary vaccination completion. ^e^The median time between the 640 dropped positives and the prior positive was 5 days (first-third quartiles: 3 to 7 days). ^f^There were 134 people with missing SVI data, 40 people with missing sex data and none with missing age data. People were allowed to contribute up to three negative tests to the control sample. If they had more than three negative tests over the study period, three tests were randomly selected. If a person had more than one negative test within a 7-day period, one test performed within that period was randomly selected. COVID-19, Coronavirus Disease 2019; RT-PCR, reverse transcription PCR; SGTF, S-gene target failure.

We excluded tests that were performed after receiving a heterologous primary vaccination (i.e., different first and second dose manufacturers) or an Ad26.COV2 vaccine dose. Additionally, we excluded tests that were performed among people who received booster doses prior to eligibility (defined as five months since second primary vaccine dose and after booster vaccination approval in the US [September 22, 2021]) [19]. We excluded tests that were performed in the 90 days after a positive SARS-CoV-2 test (rapid antigen or RT-PCR), had a positive reflex result with an inconclusive SGTF finding, were obtained from people with more than one prior SARS-CoV-2 infection or with missing confounder data, or occurred after a second booster (fourth) dose (Fig 2).

The Yale Computational Health Platform was used to extract demographic, comorbidity, COVID-19 vaccination, and SARS-CoV-2 testing data from EMR [20,21]. As an organization in EPIC’s Care Everywhere Network, YNHH’s EMR contains records from all participating health systems and organizations [22]. Additional COVID-19 vaccination records from the state vaccination registry were linked to the YNHH medical records and extracted through the same platform. The Yale Institutional Review Board approved this study and waived the need for informed consent (ID# 2000030222). This study is reported as per the Strengthening the Reporting of Observational Studies in Epidemiology (STROBE) guideline (S1 STROBE Checklist).

### Exposures

Our exposure of interest was time from completion of primary (two doses) and booster (third dose) vaccination with mRNA-1273 or BNT162b2. We stratified vaccination by time since dose (<14 days since second dose, 14 to 149 days since second dose, ≥150 days since second dose but prior to booster dose, <14 days since booster dose, 14 to 59 days since booster dose, 60 to 89 days since booster dose and ≥90 days since booster dose). Tests were further stratified by a history of a documented prior SARS-CoV-2 infection, defined as a positive RT-PCR or rapid antigen test result in the medical record ≥90 days before the included test (with testing going back to March 2020).

### Case and control definition and selection

A case of test-positive Omicron (lineage BA.1) infection was a positive SARS-CoV-2 RT-PCR test with SGTF, defined as (1) an ORF1ab Ct value of <30 and S-gene Ct–ORF1ab Ct value ≥5; or (2) ORF1ab Ct value <30 and S-gene Ct value ≥40 [18,23]. We defined eligible controls as negative SARS-CoV-2 RT-PCR test collected ≥7 days prior to a positive test or in the absence of a prior positive test and ≥7 days prior to the end of the study period (to account for test reporting delays). People were allowed to contribute both cases and controls, and our sample included all eligible cases and up to three negative tests (controls) per person during the study period. If a person had more than one negative test within a seven-day period, one random test was selected during the period as a control.

### Statistical analysis

We conducted two primary analyses, each stratified by documented prior SARS-CoV-2 infection status. First, using all identified cases and controls, we estimated vaccine effectiveness as one minus the odds ratio (OR) of vaccination among cases and controls. Under the assumption of the TNCC design, the OR from this analysis estimates the effectiveness of vaccination against infection [24]. Second, we examined whether a booster dose was associated with increased protection beyond that afforded by the primary series by comparing the odds of infection among recently boosted people (14 to 59 days after booster dose) to the odds among booster-eligible people. In alignment with CDC booster dose recommendations at the time of analysis [25], we defined booster-eligible people as people aged ≥12 years who completed their primary series ≥150 days (five months) prior to the included test and had not received a booster dose. For this analysis, we were interested in the level of protection associated with a booster dose. For that reason, we limited our analysis to booster eligible persons (aged ≥12 years) and to the first documented infection after booster dose administration by excluding case and control tests performed after the first recorded infection following booster dose administration.

As a secondary analysis, we evaluated whether the odds of infection changed over time after the administration of a booster dose by comparing the odds of infection among recently boosted people (14 to 59 days after booster dose) to the odds of infection among people who received their booster dose 60 to 89 and ≥90 days prior to testing [26]. Further, to test if changes in the odds of infection over time since booster dose receipt resulted in a loss of protection relative to booster-eligible individuals, we compared the odds of infection among people who received their booster dose 60 to 89 and ≥90 days prior to testing to the odds of infection among booster-eligible people. Since there was a limited number of boosted people with a prior SARS-CoV-2 infection (*n* = 680), the secondary analysis was restricted to cases and controls identified among people without a documented prior infection.

A mixed effects generalized additive logistic regression was used to evaluate associations. We included the following a priori selected covariates: date of test (continuous), age (continuous), sex, race/ethnicity (self-reported record in EMR), Charlson comorbidity score as of December 2020 (categorized as 0, 1–2, 3–4, 5+) [27], number of nonemergent YNHH encounters in the year prior to vaccine rollout in Connecticut (December 2020; categorized as 0, 1–2, 3–4, 5+), insurance group (uninsured, Medicaid, Medicare, other), social vulnerability index (SVI) of residential zip code (continuous), and municipality of residence (as captured in the EMR). Continuous factors were modeled using a natural spline with 3 knots, and we included a random intercept for municipality [28,29]. SVI is an estimate of a community’s vulnerability that comprises 15 factors including poverty level, access to transportation, and crowded housing [30]. To account for waning infection-mediated immunity, we included time since prior SARS-CoV-2 infection as a continuous factor in analyses limited to people with a documented prior infection. Significance was defined using an alpha of 0.05 and all tests were two-sided. All analyses were conducted in R, version 4.1.2 [31].

### Bias indicator and sensitivity analyses

To examine the extent of unmeasured confounding in our analysis, we compared the odds of infection among recently vaccinated people (1 to 13 days after first dose administration) to the odds of infection among unvaccinated people. As outlined in Hitchings and colleagues, the comparison of vaccinated and unvaccinated people during this period can be interpreted as the relative difference in infection risk between vaccinated and unvaccinated groups due to factors other than vaccination [32]. This analysis was performed among all cases and controls and among cases and controls stratified by a documented prior infection.

We performed multiple sensitivity analyses to ensure our findings were robust to alternative study design, data cleaning, and modeling assumptions. Specifically, we tested the robustness of our findings to the following scenarios: 1:1 matching with replacement, exclusion of heterologous booster doses, inclusion of tests among people with more than one documented prior infection, exclusion of discordant test results, inclusion of positive TaqPath results with inconclusive SGTF (included as negative tests), and inclusion of all controls. To examine if the temporality of prior SARS-CoV-2 infection and vaccinations impacted estimates of vaccine effectiveness among people with a documented prior infection, we conducted an analysis where we excluded tests performed among people whose prior SARS-CoV-2 infection occurred after the first dose of primary vaccination. Additionally, to evaluate whether waning of protection associated with primary vaccination influenced the risk comparisons between boosted and booster-eligible people, we conducted sensitivity analyses that were restricted to tests collected among people who completed primary vaccination ≥150 days prior to testing and adjusted for time since completion of primary vaccination. For a detailed description, see Supporting information S1 Text.

## Results

### Study population

Between November 1, 2021, and April 30, 2022, we identified 174,259 SARS-CoV-2 tests performed with the TaqPath assay on samples obtained from 121,967 unique people in the YNHH system (Fig 2). The first SGTF-defined Omicron (lineage BA.1) infection in the study population was identified on November 11, 2021 (Fig 1). Of the 152,109 eligible tests, 11,307 were identified as Omicron (lineage BA.1) infections (cases), contributed from 11,307 unique people. From the 140,802 negative RT-PCRs, we randomly selected up to three negative tests (controls) per person, resulting in 130,041 controls (Fig 2). The average number of controls per person were similar among people who contributed a case (1.47, SD: 0.75) and people who did not contribute a case (1.31, SD: 0.62).

Cases and controls had similar characteristics with respect to age, sex, SVI of residential zip code, and Charlson comorbidity score (Table 1). However, a larger proportion of Omicron (lineage BA.1) cases occurred among non-Hispanic black people (16.1% versus 10.2% in controls) and those who were uninsured (11.2% versus 7.6% in controls) or received Medicaid (22.2% versus 13.3% in controls). Among boosted people, the median time between booster vaccination and testing was similar for cases (57 days [first-third quartile: 29 to 83 days]) and controls (47 days [first-third quartile: 26 to 76 days]). Among cases and controls, 5.9% (672/11,307) and 8.1% (10,473/130,041) had a prior SARS-CoV-2 infection. The time between a documented prior infection and testing was similar for cases (375 days [first-third quartile: 296 to 420 days]) and controls (332 days [first-third quartile: 256 to 391 days]; Table 1) The vast majority (98.7%, 11,002/11,145) of prior documented infections occurred before the emergence of Omicron in November 2021 and 99.6% (11,097/11,145) occurred before Omicron became the dominate circulating variant in Connecticut [17,33].

**Table 1:** Characteristics of SARS-CoV-2 tests included as cases or controls between November 1, 2021 and April 30, 2022.

	Case^a^	Control^a^
Characteristic	(*N* = 11,307)^b^	(*N* = 130,041)^b^
Age in years [Median (p25-p75^b^)]	35 (21, 50)	39 (22, 56)
Sex [N^b^ (%)]		
Female	6,243 (55.2%)	76,062 (58.5%)
Male	5,064 (44.8%)	53,979 (41.5%)
Race/Ethnicity [N^b^ (%)]		
Black or African American	1,815 (16.1%)	13,307 (10.2%)
Hispanic or Latino	2,325 (20.6%)	15,377 (11.8%)
Other/Unknown	1,565 (13.8%)	19,004 (14.6%)
White	5,602 (49.5%)	82,353 (63.3%)
Social vulnerability index (SVI) Median (p25-p75^b^)]	0.5 (0.5, 0.5)	0.5 (0.5, 0.5)
Insurance group [N^b^ (%)]		
Uninsured	1,267 (11.2%)	9,911 (7.6%)
Medicaid	2,514 (22.2%)	17,240 (13.3%)
Medicare	318 (2.8%)	6,670 (5.1%)
Other	7,208 (63.7%)	96,220 (74.0%)
Nonemergent healthcare visits^c^ [Median (p25-p75^b^)]	0.0 (0.0, 5.0)	0.0 (0.0, 5.0)
Charlson comorbidity score^d^ [Median (p25-p75^b^)]	0.0 (0.0, 0.0)	0.0 (0.0, 1.0)
Documented prior SARS-CoV-2 infection^e^ [N^b^ (%)]		
Yes	672 (5.9%)	10,473 (8.1%)
No	10,635 (94.1%)	119,568 (91.9%)
Vaccination status at time of testing [N^b^ (%)]		
Unvaccinated	5,760 (50.9%)	62,437 (48.0%)
Incomplete primary vaccination (<14 days after second dose)	753 (6.7%)	9,367 (7.2%)
Complete primary vaccination		
14–149 days after second dose	511 (4.5%)	4,492 (3.5%)
≥150 days after second dose (pre-booster dose)	3,625 (32.1%)	41,794 (32.1%)
Booster vaccination		
<14 days after booster (third) dose	72 (0.6%)	1,378 (1.1%)
≥14 days after booster (third) dose	586 (5.2%)	10,573 (8.1%)
SARS-CoV-2 testing^f^		
Days after second dose [Median (p25-p75^b^)]	247 (217, 279)	240 (203, 279)
Days after booster (third) dose [Median (p25-p75^b^)]	57 (29, 83)	47 (26, 76)
Days after documented prior SARS-CoV-2 infection [Median (p25-p75^b^)]	375 (296, 420)	332 (256, 391)

^a^Participants allowed to contribute both cases and up to three control tests, cases were limited to Omicron (lineage BA.1) cases defined as presence of SGTF (S-gene target failure).

^b^p25-p75 refers to the first and third quartile; N refers to the number of tests.

^c^Number of nonemergent visits to YNHH (Yale New Haven Health) in the year (December 2, 2019-December 1, 2020) prior to initiation of COVID-19 vaccination at YNHH.

^d^Score as of December 2020.

^e^Documented prior infections defined as positive RT-PCR or rapid antigen test performed ≥90 days prior to the index test.

^f^SARS-CoV-2 testing: 51,265 tests were collected after the second dose; 12,609 tests were collected after a booster dose; 11,145 tests were collected after a documented prior infection.

### Risk of Omicron (lineage BA.1) infection among boosted and booster-eligible people

**Fig 3:**
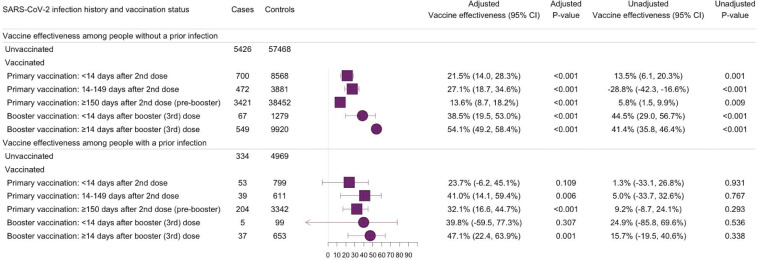
Effectiveness of primary and booster vaccination with COVID-19 mRNA vaccines against SARS-CoV-2 Omicron (BA.1 lineage) variant infections, stratified by the history of a documented prior SARS-CoV-2 infection. Forest plot depicting vaccine effectiveness against Omicron (lineage BA.1) infections for US-approved mRNA vaccines (BNT162b2 and mRNA-1273) among people with and without a documented prior infection. A documented prior infection was defined as a positive RT-PCR or rapid antigen test at least 90 days before the included test. Omicron (lineage BA.1) infection was defined as the presence of SGTF defined as ORF1ab Ct <30 and S-gene–ORF1ab ≥ 5, or ORF1ab <30 and S-gene ≥ 40. Vaccine effectiveness was estimated as one minus the OR from a model adjusted for date of test, age, sex, race/ethnicity, Charlson comorbidity score, number of nonemergent visits in the year prior to the vaccine rollout in Connecticut, insurance status, municipality, and SVI of residential zip code in all analyses and time between testing and last documented prior infection in analyses of people with a documented prior infection. COVID-19, Coronavirus Disease 2019; OR, odds ratio; RT-PCR, reverse transcription PCR; SARS-CoV-2, Severe Acute Respiratory Syndrome Coronavirus 2; SGTF, S-gene target failure; SVI, social vulnerability index.

During the period prior to booster eligibility (14 to 149 days after second dose), the effectiveness of primary mRNA vaccination against Omicron (lineage BA.1) infection was 41.0% (95% CI: 14.1% to 59.4%, *p* 0.006) and 27.1% (95% CI: 18.7% to 34.6%, *p* < 0.001) for people with and without a documented prior infection, respectively. During the period of booster eligibility (150+ days after second dose), the effectiveness of primary vaccination was 32.1% (95% CI: 16.6% to 44.7%, *p* < 0.001) for people with and 13.6% (95% CI: 8.7% to 18.2%, *p* < 0.001) for people without a documented prior infection. Vaccine effectiveness in the period 14 to 59 days after a booster dose was 47.1% (95% CI: 22.4% to 63.9%, *p* 0.001) and 54.1% (95% CI: 49.2% to 58.4%, *p* < 0.001) for people with and without a documented prior infection, respectively (Fig 3).

Among people without a documented prior infection, the odds of Omicron (lineage BA.1) infection were 0.54 (95% CI: 0.49 to 0.59, *p* < 0.001) times lower for boosted than booster-eligible people. Conversely, the odds of infection did not differ significantly between boosted and booster-eligible people with a documented prior infection (OR: 0.79, 95% CI: 0.54 to 1.16, *p* 0.222). Ten controls (negative RT-PCR tests) were collected among people with a documented prior infection that occurred after they received their booster dose and were excluded from this analysis (Table 2).

**Table 2:** Risk of SARS-CoV-2 Omicron (BA.1 lineage) variant infection among people who received booster vaccination relative to booster-eligible people, according to history of a documented prior SARS-CoV-2 infection.

			Adjusted^b^		Unadjusted	
Prior SARS-CoV-2 infection history and vaccination status^a^	Cases	Controls	Odds ratio^c^	*P* value	Odds ratio^c^	*P* value
**With a documented prior infection** ^**d**^						
Booster-eligible, ≥150 days after second dose (pre-booster dose)^e^	204	3,342	-	-	-	-
Boosted, ≥14 days after booster (third) dose^f^	37	643	0.79 (0.54, 1.16)	0.222	0.94 (0.66, 1.35)	0.748
**Without a documented prior infection** ^**d**^						
Booster-eligible, ≥150 days after second dose (pre-booster dose)^e^	3,421	38,446	-	-	-	-
Boosted, ≥14 days after booster (third) dose^f^	549	9,920	0.54 (0.49, 0.59)	<0.001	0.62 (0.57, 0.68)	<0.001

^a^Due to booster eligibility at the time of the analysis, this analysis was limited to persons 12 years or greater, resulting in the exclusion of 6 booster-eligible controls.

^b^Adjusted for date of test, age, sex, race/ethnicity, insurance, Charlson Comorbidity Score, SVI (Social Vulnerability Index) of zip code, municipality, and number of nonemergent visits during the year prior to vaccine rollout in Connecticut (December 2, 2019 and December 1, 2020) in all analyses and time between testing and last a documented prior infection in analyses of people with a documented prior infection.

^c^Point estimate (95% confidence interval).

^d^Documented prior infection defined as a positive RT-PCR or rapid antigen test at least 90 days prior to included test.

^e^Limited to booster-eligible people, booster-eligible defined as primary series recipients aged 12 years or more who completed their primary series (2 doses) 150+ days prior to the test and were yet to receive a booster (third) dose; 150 days was selected as it reflects the CDC booster recommendations at the time of manuscript submission.

^f^Ten tests (all controls) were collected among people with a documented prior infection that occurred following their booster dose and were removed from this analysis.

In the secondary analysis that was restricted to people without a documented prior infection, the odds of Omicron (lineage BA.1) infection increased over time since booster vaccination and were significantly higher 90+ days after a booster dose relative to the period 14 to 59 days after the dose (OR: 1.78, 95% CI: 1.41 to 2.25, *p* < 0.001) (S1 Table). Yet, the odds of infection among boosted people 90+ days after the booster dose was lower than the odds among booster-eligible people (OR: 0.80, 95% CI: 0.66 to 0.98, *p* 0.030) (Table 3).

**Table 3:** Risk of SARS-CoV-2 Omicron (BA.1 lineage) variant infection among people who received booster vaccination relative to booster-eligible people^a^, according to time after receiving a booster vaccine dose.

			Adjusted^b^		Unadjusted	
Vaccine status at testing	Case	Control	Odds ratio^c^	*P* value	Odds Ratio^c^	*P* value
Primary Series: 150+ days after second dose (pre-booster dose)	3,421	38,446	-	-	-	-
Boosted: 14–59 days after booster (third) dose	263	5,637	0.45 (0.39, 0.52)	<0.001	0.52 (0.46, 0.60)	<0.001
Boosted: 60–89 days after booster (third) dose	164	2,313	0.56 (0.47, 0.66)	<0.001	0.80 (0.68, 0.94)	0.006
Boosted: 90+ days after booster (third) dose	122	1,970	0.80 (0.66, 0.98)	0.030	0.70 (0.58, 0.84)	<0.001

^a^Limited to people without prior documented SARS-CoV-2 infections; limited to booster-eligible people, booster-eligible defined as primary series recipients aged 12 years or more who completed their primary series (2 doses) 150+ days prior to the test and were yet to receive a booster (third) dose; 150 days was selected as it reflects the CDC booster recommendations at the time of manuscript submission.

^b^Adjusted for date of test, age (in years), sex, race/ethnicity, insurance, comorbidity (Charlson Score), SVI (Social Vulnerability Index) of zip code, presence of a documented prior infection, municipality, and number of nonemergent visits during the year prior to vaccine rollout in Connecticut (December 2, 2019 and December 1, 2020).

^c^Point estimate (95% confidence interval).

### Bias indicator and sensitivity analyses

We estimated the reduction in infection risk during the 13 days after first dose administration as an indicator of potential bias. From this analysis, we observe some difference in risk among our groups during this period but with low precision (all tests: 23.2%, 95% CI: −8.8% to 45.8%, *p* 0.138) (S2 Table).

In sensitivity analyses, the effectiveness of booster vaccination against Omicron (lineage BA.1) infection (≥14 days after the booster dose) ranged from 35.7% to 50.7% for people with a documented prior infection and from 51.3% to 55.9% for people without a documented prior infection (Figs A-H in S1 Text). Compared with the primary analysis, we observed a smaller sample size and lower precision (wider CIs) from our matched (1:1 with replacement) analysis (matched analysis CIs were ≥3.4 units wider than primary analysis; Fig A in S1 Text). Following the exclusion of tests collected among people whose documented prior infection occurred after their first vaccine dose, we observed nonsignificantly lower effectiveness estimates for primary (14 to 149 days after second dose: 39.5%, 95% CI: 12.0% to 58.4%, *p* 0.009) and booster (≥14 days after booster dose: 45.7%, 95% CI: 20.3% to 62.9%, *p* 0.002) vaccination (Fig H in S1 Text). Adjusting for time between testing and completion of primary vaccination did not significantly alter the estimated association between booster doses and Omicron (lineage BA.1) infections among people with a documented prior infection (OR: 0.75, 95% CI: 0.50 to 1.13, *p* 0.165) or without a documented prior infection (OR: 0.57, 95% CI: 0.51 to 0.63, *p* < 0.001) (Table A in S1 Text).

## Discussion

Leveraging data from a large population of Connecticut and nearby state residents, we examined the effectiveness of primary and booster mRNA vaccination against Omicron (lineage BA.1) infections among vaccine-eligible people with and without a documented prior infection. We found that primary vaccination was associated with significant but low levels of protection among people with and without a documented prior infection. While booster vaccination was associated with protection beyond that afforded by the primary series in people without a documented prior infection, we did not identify a significant increase in protection among people with a documented prior infection.

Contrary to the findings of Shrestha and colleagues [12], our analysis, which ascertained Omicron (lineage BA.1) infection in cases by the presence of SGTF and had increased precision for vaccine effectiveness, found that primary vaccination was associated with a significant reduction in the risk (41.0%, 95% CI: 14.1% to 59.4%, *p* 0.006) of Omicron (lineage BA.1) infection among people with a documented prior infection. Though we found the level of protection afforded by primary vaccination to be low, our findings suggest that primary vaccination may be warranted regardless of documented prior infection status.

Our estimate of booster vaccination effectiveness among people with a documented prior infection had a smaller sample size and reduced precision (37 cases, 95% CI: 22.4% to 63.9%) compared to the estimate for people without a documented prior infection (549 cases, 95% CI: 49.2% to 58.4%). However, in a parallel analysis, the odds of infection did not differ significantly (OR: 0.79, 95% CI: 0.54 to 1.16, *p* 0.222) between boosted and booster-eligible people with a documented prior SARS-CoV-2 infection but differed significantly for people without a prior documented infection (OR: 0.54, 95% CI: 0.49 to 0.59, *p* < 0.001).

While differences in the estimates for people with and without a documented prior infection may be partially driven by differences in care-seeking behaviors, together, these findings suggest that the benefit of booster doses among booster-eligible individuals without a documented prior infection may be greater than the benefit among booster-eligible people with a documented prior infection. They further suggest that boosters may not confer additional protection beyond that afforded by primary vaccination among previously infected people. However, another study looking at the same question among staff and residents of correctional facilities found that booster doses provided significant additional protection among people with prior infections [34]. While their population is highly specific, these alternative findings, along with the reduced precision of our estimates among people with a documented prior infection, indicate the need for additional research examining the benefits of booster doses within this subpopulation.

In alignment with prior studies [1,8,35], we found the risk of Omicron (lineage BA.1) infection among boosted people without a documented prior infection increased significantly three months after booster dose administration. However, the odds of infection among boosted people remained significantly lower than the odds among booster-eligible people (OR: 0.80, 95% CI: 0.66 to 0.98, *p* 0.030). Thus, even with the decline in protection, booster vaccination appears to provide additional protection beyond that conferred by primary vaccination in people without a documented prior SARS-CoV-2 infection.

Weekly testing for certain unvaccinated professionals, such as employees of healthcare facilities that accept Medicare and/or Medicaid or Connecticut state employees, was required by the state and federal government during the study period [36,37]. Because such requirements resulted in increased testing among unvaccinated but not vaccinated persons, our vaccine effectiveness estimates are likely conservative. Further, this may explain the higher proportion of unvaccinated people we observed compared to the state vaccination coverage (unvaccinated cases: 50%, unvaccinated controls; 48%, state vaccination coverage [April 27, 2022]: range between 48.8% for people 5 to 11 years old and 100% for people 65 to 74 years old) [38]. However, the bias introduced by required testing does not extend to the comparisons among vaccinated groups and our findings comparing boosted to booster-eligible people are likely to be unaffected by testing policies.

Our analysis was limited to a population of Connecticut residents and was reliant on medical record data that is subject to misclassification. In the place of whole genome sequence data, we used SGTF status to ascertain Omicron infections as cases. SGTF as a proxy for Omicron status has been widely used during the Omicron epidemic wave in the winter of 2021 to 2022 and has been recommended as an indicator of Omicron lineage BA.1 infection by WHO [23]. However, the use of this variant designation resulted in our analysis being limited to Omicron lineage BA.1 and may have resulted in the exclusion of coinfections (infection with both Omicron lineage BA.1 and another SARS-CoV-2 variant without SGTF) that did not meet our outcome definition. TaqPath testing was primarily employed in the YNHH outpatient setting and thus disproportionately represents mild cases of SARS-CoV-2 infection. However, our SGTF definition required Ct values <30, which may be biased towards more severe illness. These sampling restrictions may impact the generalizability of our findings.

We did not have adequate sample to evaluate the level of protection conferred by two or more documented prior infections (*n* = 77) or to examine vaccine effectiveness among people who received two booster doses. We expect a proportion of prior SARS-CoV-2 infections may have gone undetected and that ascertainment of documented prior infection history may be subject to misclassification. Nevertheless, our analyses that are restricted to people with a documented prior infection are unaffected by such misclassification. For the OR comparing vaccination between test-positive cases and test-negative controls to equal the vaccine effectiveness against infection, the following assumptions must hold: (1) vaccination must be uncorrelated with exposure or susceptibility to infection; (2) testing behaviors must be uncorrelated with vaccination status; and (3) vaccination must confer all-or-nothing protection. Despite accounting for numerous factors that either confound the relationship between vaccination and exposure or between vaccination and testing (including recent healthcare utilization and SVI), residual confounding and differential healthcare-seeking behavior may persist. However, our bias indicator (the association between recent vaccination [1 to 13 days after first dose administration] and infection) did not indicate any sizeable bias. Though we could not test if the mechanism of protection was all-or-nothing, this assumption is fundamental to vaccine effectiveness analyses (including randomized control trials) and would remain applicable under an alternative design approach [24].

Prior theoretical work has cautioned that if vaccination reduces disease severity, the results of a TNCC study may be subject to bias. While we did not have adequate sample to restrict our analysis to symptomatic infections (1,584 cases) and reduce the potential for this bias, the small sample size stems from the mild disease presentation in our examined sample (as discussed previously). If we assume that vaccination reduces severity, this overrepresentation of mild infections would likely result in a disproportionately high number of infections among vaccinated people and could result in conservative effectiveness estimates. Finally, the analyses were not powered to test associations for severe COVID-19; we therefore cannot exclude that booster vaccination may increase protection against such outcomes in people with a prior SARS-CoV-2 infection, as others have concluded [39].

Primary vaccination with two COVID-19 mRNA vaccine doses provided significant but limited protection against Omicron (lineage BA.1) infection among people with and without a prior SARS-CoV-2 infection. While booster vaccination resulted in additional protection beyond that afforded by the primary vaccination among people without a documented prior infection, it did not result in additional protection among previously infected people. These findings support primary vaccination regardless of prior infection history but suggest that a person’s history of prior SARS-CoV-2 infection may affect the relative benefit of booster doses.

## Supporting information

S1 STROBE Checklist Filled out STROBE checklist. (DOCX)

S1 Text Sensitivity analyses. Supporting information holding the description and results from the sensitivity analyses discussed in the manuscript. Fig A in S1 Text. Forest plot of vaccine effectiveness from matched analysis, stratified by the history of a prior SARS-CoV-2 infection. Fig B in S1 Text. Number of events among boosted individual by primary and booster dose vaccine brand. Fig C in S1 Text. Forest plot of vaccine effectiveness exclusive of test among people with a heterologous booster dose, stratified by the history of a prior SARS-CoV-2 infection. Fig D in S1 Text. Forest plot of vaccine effectiveness following the inclusion of tests collected after multiple prior positives, stratified by the history of a prior SARS-CoV-2 infection. Fig E in S1^1^ Text. Forest plot of vaccine effectiveness exclusive of discordant Thermo Fisher and reflex resulted tests, stratified by the history of a prior SARS-CoV-2 infection. Fig F in S1 Text. Forest plot of vaccine effectiveness following the inclusion of inconclusive SGTF as controls, stratified by the history of a prior SARS-CoV-2 infection. Fig G in S1 Text. Forest plot of vaccine effectiveness inclusive of all controls, stratified by the history of a prior SARS-CoV-2 infection. Fig H in S1 Text. Forest plot of vaccine effectiveness following the exclusion of tests among people with prior infections after a vaccine dose, stratified by the history of a prior SARS-CoV-2 infection. Table A in S1 Text. Association between booster dose and risk of SARS-CoV-2 Omicron variant infection by prior SARS-CoV-2 infection history.
(DOCX)

S1 Table Duration of booster dose protection. Risk of SARS-CoV-2 Omicron variant infection ≥60 days after booster vaccination relative to people who received a booster 14–59 days prior to testing.
(XLSX)

S2 Table Bias indicator. Results from the bias indicator analysis described in the manuscript.
(XLSX)

## Abbreviations

CI: confidence interval
COVID-19: Coronavirus Disease 2019
EMR: electronic medical record
OR: odds ratio
RT-PCR: reverse transcription PCR
SARS-CoV-2: Severe Acute Respiratory Syndrome Coronavirus 2
SGTF: S-gene target failure
SVI: social vulnerability index
TNCC: test-negative case–control
YNHH: Yale New Haven Health System
