# Supplementary material for: Association between primary or booster COVID-19 mRNA vaccination and Omicron lineage BA.1 SARS-CoV-2 infection in people with a prior SARS-CoV-2 infection: A test-negative case–control analysis
Source: PLoS Med. 2022 Dec 1;19(12):e1004136. doi: 10.1371/journal.pmed.1004136 (PMC9714718; doi:10.1371/journal.pmed.1004136)
Supplement: S1 Text — Supporting information holding the description and results from the sensitivity analyses discussed in the manuscript. Fig A in S1 Text. Forest plot of vaccine effectiveness from matched analysis, stratified by the history of a prior SARS-CoV-2 infection. Fig B in S1 Text. Number of events among boosted individual by primary and booster dose vaccine brand. Fig C in S1 Text. Forest plot of vaccine effectiveness exclusive of test among people with a heterologous booster dose, stratified by the history of a prior SARS-CoV-2 infection. Fig D in S1 Text. Forest plot of vaccine effectiveness following the inclusion of tests collected after multiple prior positives, stratified by the history of a prior SARS-CoV-2 infection. Fig E in S11 Text. Forest plot of vaccine effectiveness exclusive of discordant Thermo Fisher and reflex resulted tests, stratified by the history of a prior SARS-CoV-2 infection. Fig F in S1 Text. Forest plot of vaccine effectiveness following the inclusion of inconclusive SGTF as controls, stratified by the history of a prior SARS-CoV-2 infection. Fig G in S1 Text. Forest plot of vaccine effectiveness inclusive of all controls, stratified by the history of a prior SARS-CoV-2 infection. Fig H in S1 Text. Forest plot of vaccine effectiveness following the exclusion of tests among people with prior infections after a vaccine dose, stratified by the history of a prior SARS-CoV-2 infection. Table A in S1 Text. Association between booster dose and risk of SARS-CoV-2 Omicron variant infection by prior SARS-CoV-2 infection history. (DOCX) [file pmed.1004136.s002.docx]

**S1 Supplemental Information:**

**Sensitivity Analyses:**

**Fig A: Forest Plot of Vaccine Effectiveness from Matched Analysis, Stratified by the History of a Prior SARS-CoV-2 Infection**

**Fig B: Number of Events Among Boosted Individual by Primary and Booster Dose Vaccine Brand**

**Fig C: Forest Plot of Vaccine Effectiveness Exclusive of Test Among People with a Heterologous Booster Dose, Stratified by the History of a Prior SARS-CoV-2 Infection**

**Fig D: Forest Plot of Vaccine Effectiveness Following the Inclusion of Tests Collected After Multiple Prior Positives, Stratified by the History of a Prior SARS-CoV-2 Infection**

**Fig E: Forest Plot of Vaccine Effectiveness Exclusive of Discordant Thermo Fisher and Reflex Resulted Tests, Stratified by the History of a Prior SARS-CoV-2 Infection**

**Fig F: Forest Plot of Vaccine Effectiveness Following the Inclusion of Inconclusive SGTF as Controls, Stratified by the History of a Prior SARS-CoV-2 Infection**

**Fig G: Forest Plot of Vaccine Effectiveness Inclusive of all Controls, Stratified by the History of a Prior SARS-CoV-2 Infection**

**Fig H: Forest Plot of Vaccine Effectiveness Following the Exclusion of Tests Among People with Prior Infections After a Vaccine Dose, Stratified by the History of a Prior SARS-CoV-2 Infection**

**Table A: Association Between Booster Dose and Risk of SARS-CoV-2 Omicron Variant Infection by Prior SARS-CoV-2 Infection History**

*Matching (1:1 with replacement):* To allow for a single analytic sample from which to perform our analyses, we did not perform a match for our primary analysis. However, our fully adjusted (un-matched) model may suffer from positivity violations. To test if matching resulted in increased precision, we performed a 1:1 match with replacement on date of test (+/- 7 day), municipality, and presence of prior infections. Of the 11307 cases in the primary study, 11161 (98.7%) matched. The results of this analysis are below.

**Fig A: Forest Plot of Vaccine Effectiveness from Matched Analysis, Stratified by the History of a Prior SARS-CoV-2 Infection**

A. Adjusted


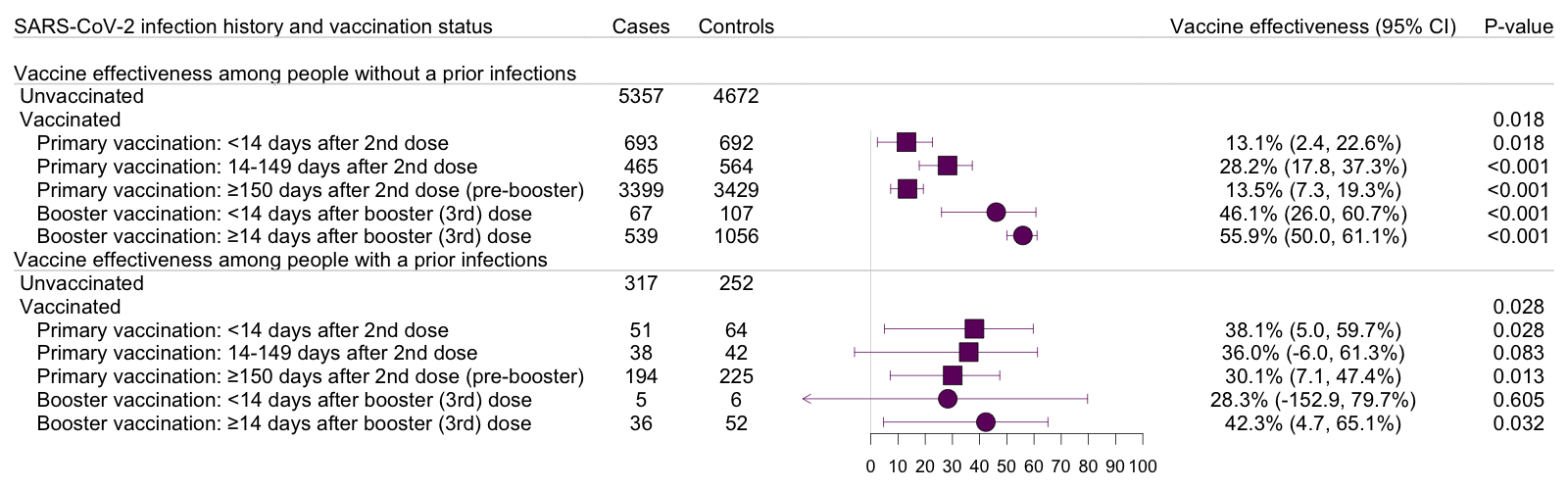


B. Unadjusted


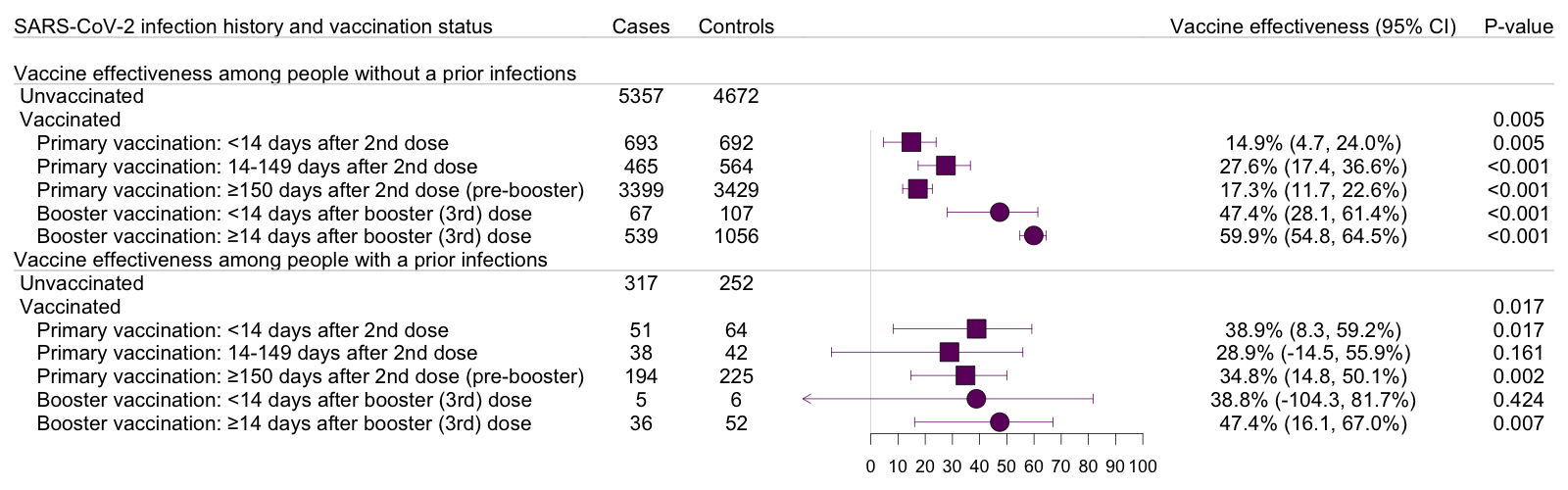


*Homologous booster doses:* We allowed for the inclusion of heterologous mRNA (mRNA-1273 and BNT162b2) booster doses (ex. Moderna booster doses for people who received a primary Pfizer dose). While most people received homologous doses, <10% received a booster dose that did not align with their primary series. Four tests were collected among people who received a primary series mRNA and a booster J&J dose and were not considered in any analysis.

**Fig B: Number of Events Among Boosted Individual by Primary and Booster Dose Vaccine Brand**

*
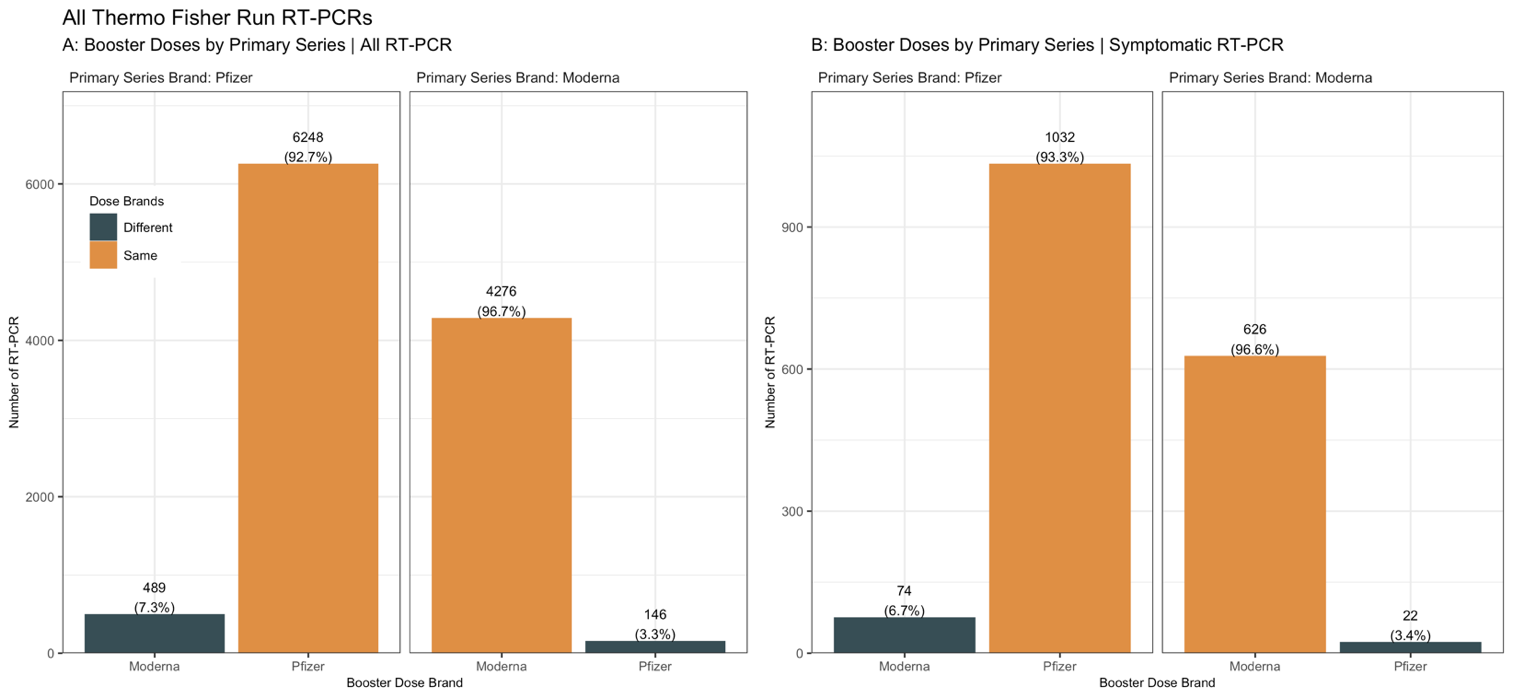
*

To test if discordant vaccine administration impacted our results, we restricted our sample to people who had homologous booster doses.

**Fig C: Forest Plot of Vaccine Effectiveness Excluding Tests Collected Among People with a Heterologous Booster Dose, Stratified by the History of a Prior SARS-CoV-2 Infection**

A. Adjusted


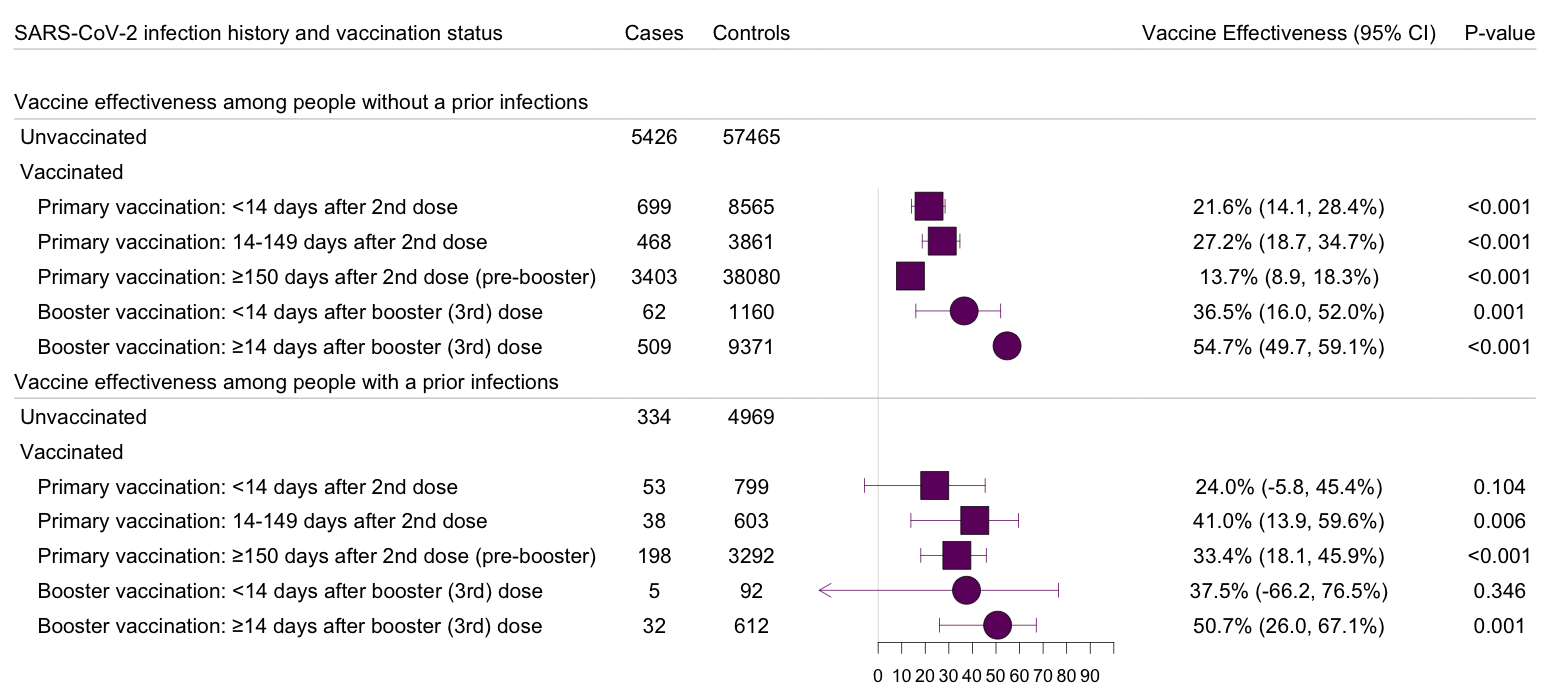


B. Unadjusted


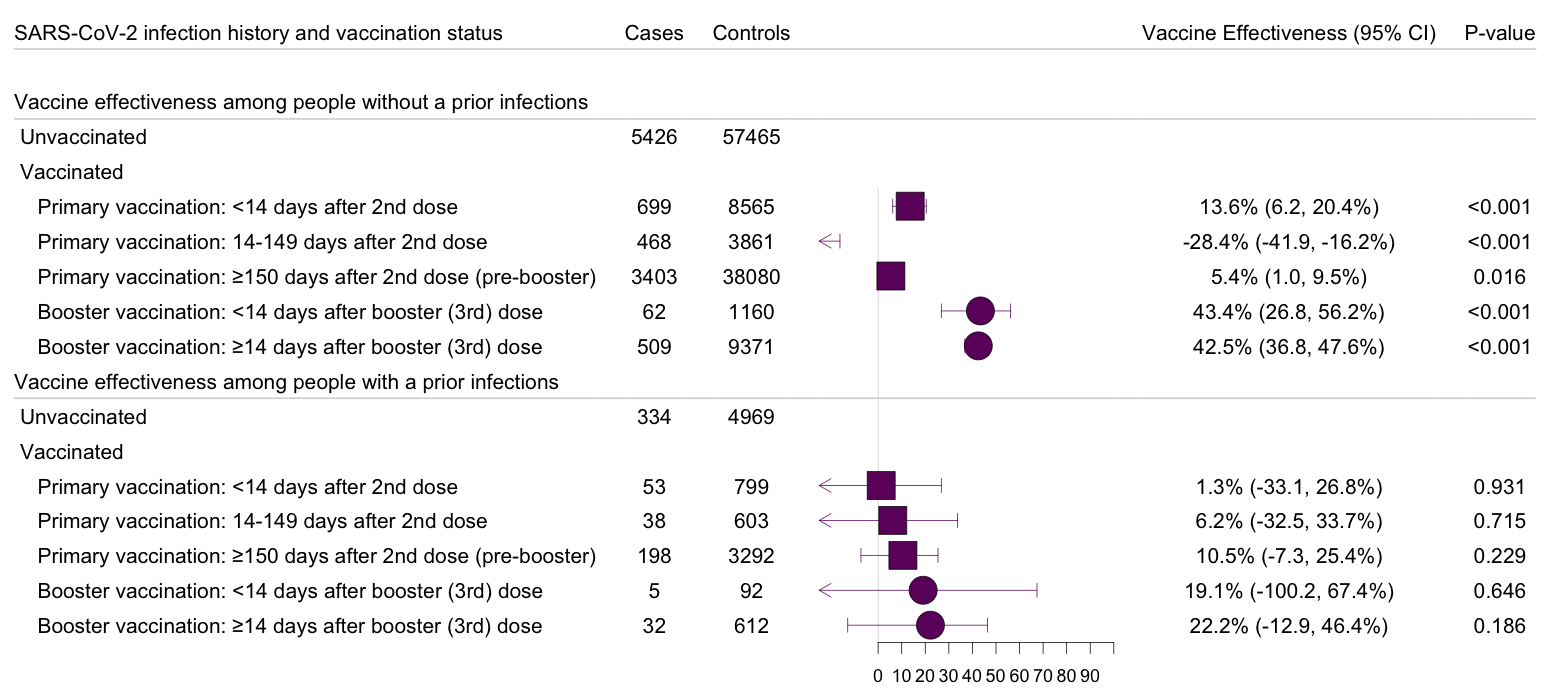


*Inclusion of tests collected among people with more than one prior documented infection:* In the primary analysis, we excluded tests collection after multiple prior positives. While this allowed us to estimate vaccine effectiveness among people with a prior infection, it was limited in its scope. Here we remove that restriction.

**Fig D: Forest Plot of Vaccine Effectiveness Including Tests Collected After Multiple Prior Documented Infections, Stratified by the History of a Prior SARS-CoV-2 Infection**

A. Adjusted


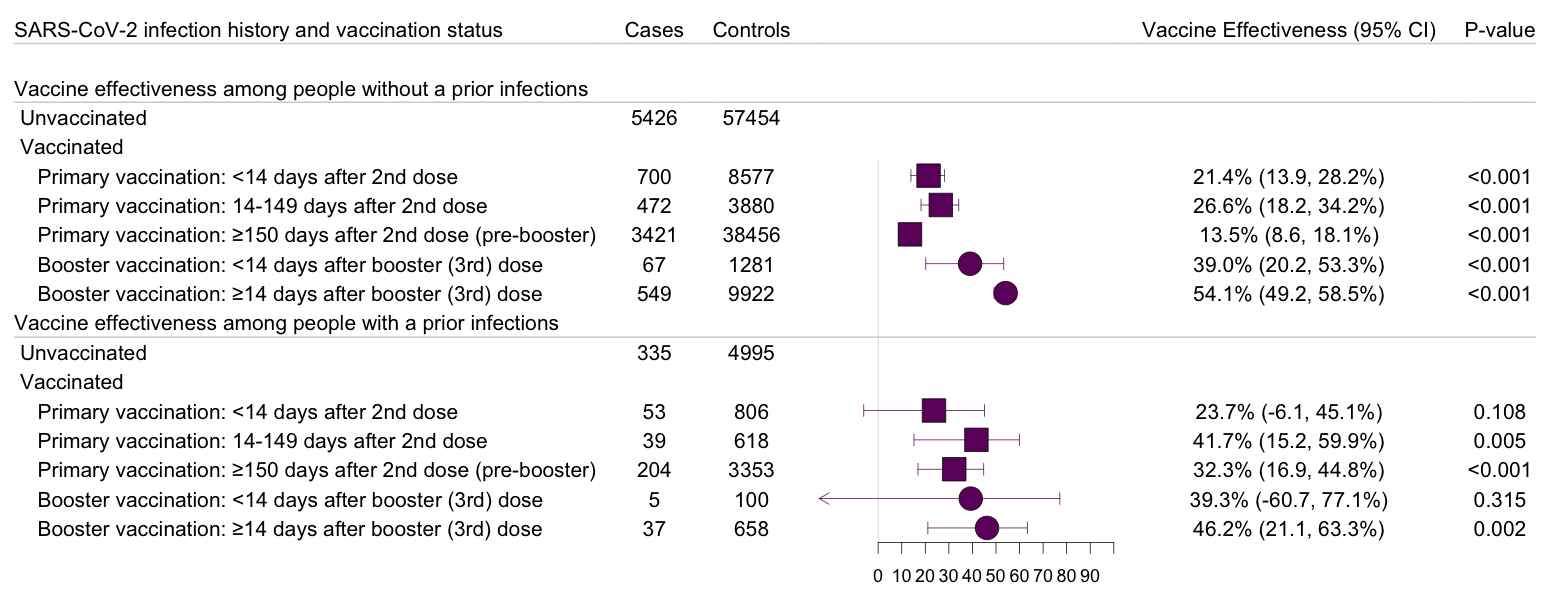


B. Unadjusted


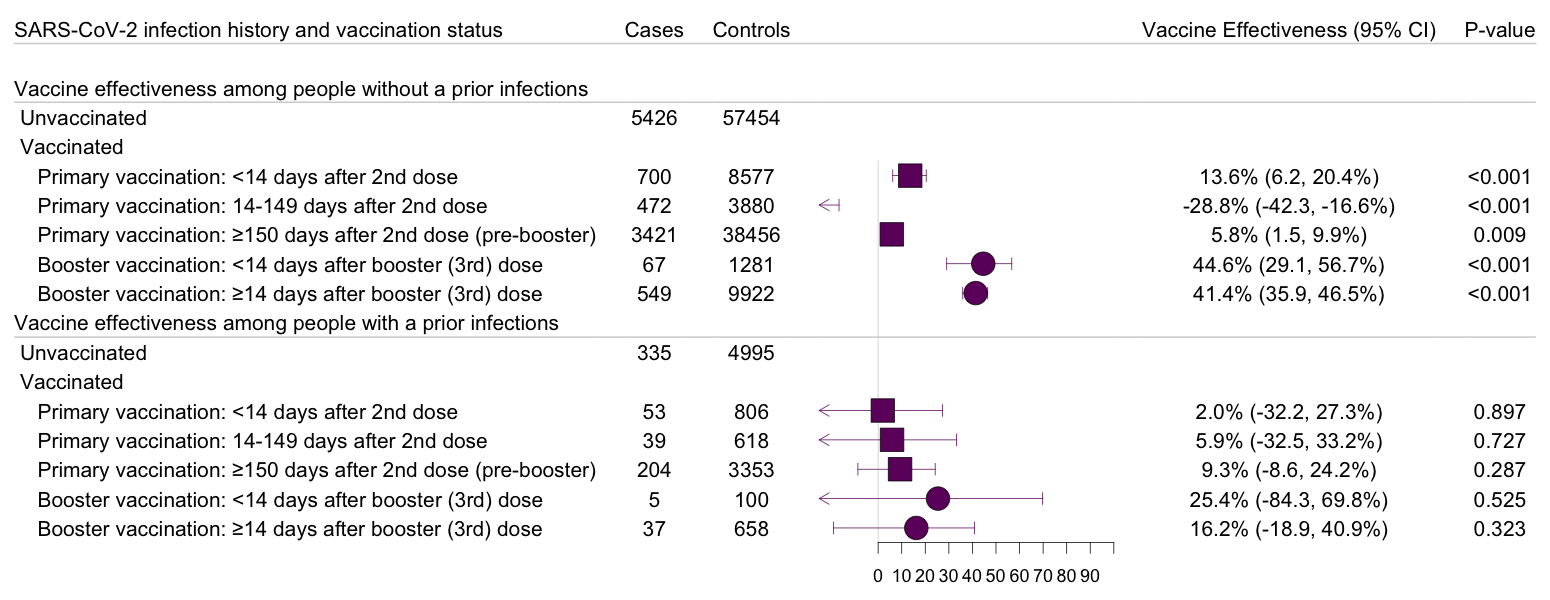


*Exclude Discordant Thermo Fisher and Reflex Tests:* In the primary analysis, we defined test positivity based on the results of the reflex test. Below are the vaccine effectiveness estimates when these tests were dropped.

**Fig E: Forest Plot of Vaccine Effectiveness Excluding Discordant Thermo Fisher and Reflex Results, Stratified by the History of a Prior SARS-CoV-2 Infection**

A. Adjusted


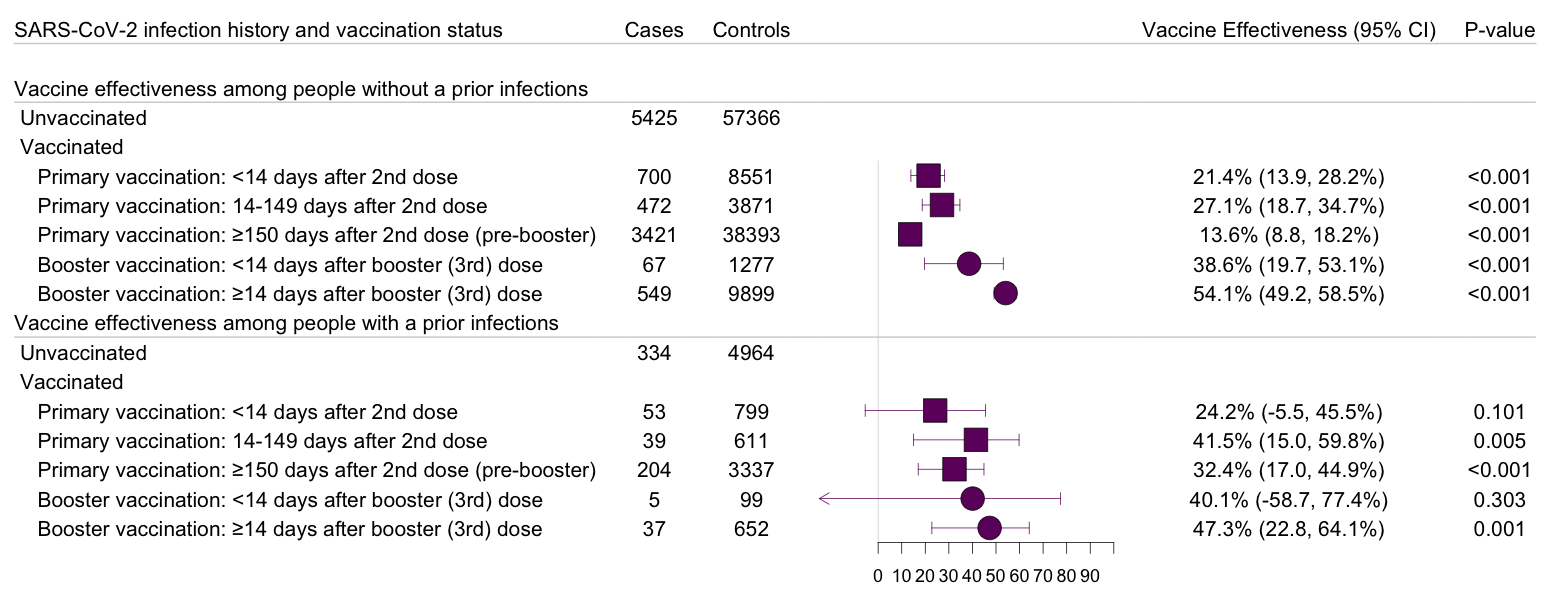


B. Unadjusted


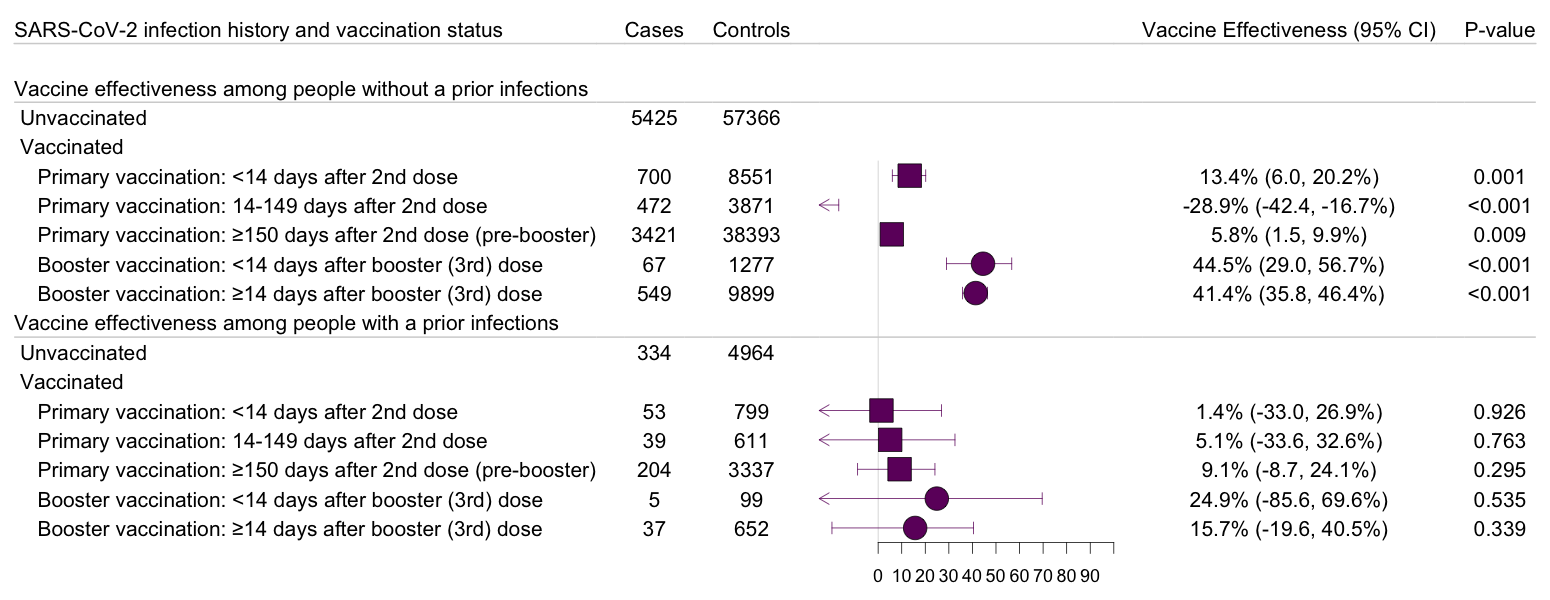


*Positive results with inconclusive SGTF listed as negative:* We defined presence of SGTF as [ORF1ab < 30] And [S Gene – ORF1ab >= 5] OR [ORF1ab < 30] And [S Gene >= 40]. If a sample was reported as positive but the ORF1ab read was > 30, SGTF was considered inconclusive. In the primary analysis, we excluded these positive results with inconclusive SGTF findings. This resulted in us having a clear variant designation for all positive RT-PCRs. Another way to handle such findings is to classify the sample as negative.^38^ Below are the vaccine effectiveness estimates when these tests were included as negatives.

**Fig F: Forest Plot of Vaccine Effectiveness Including Inconclusive SGTF Tests as Controls, Stratified by the History of a Prior SARS-CoV-2 Infection**

A. Adjusted

*
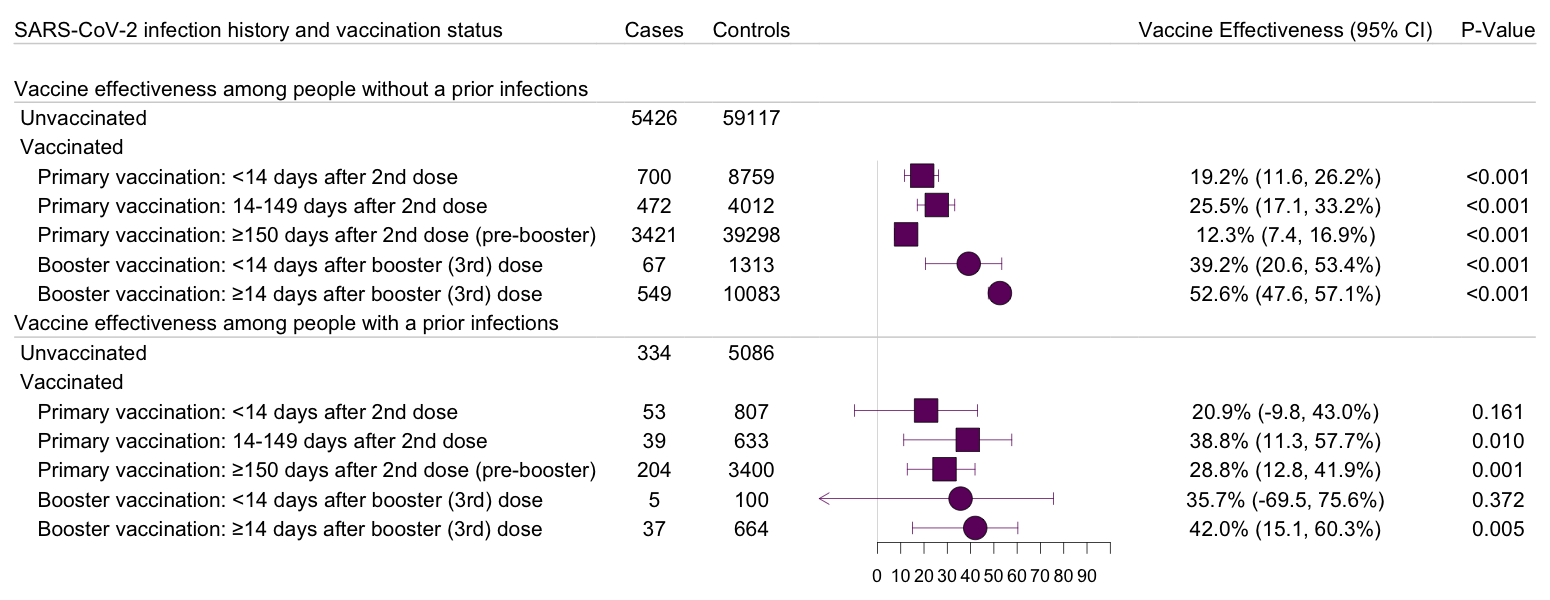
*

B. Unadjusted


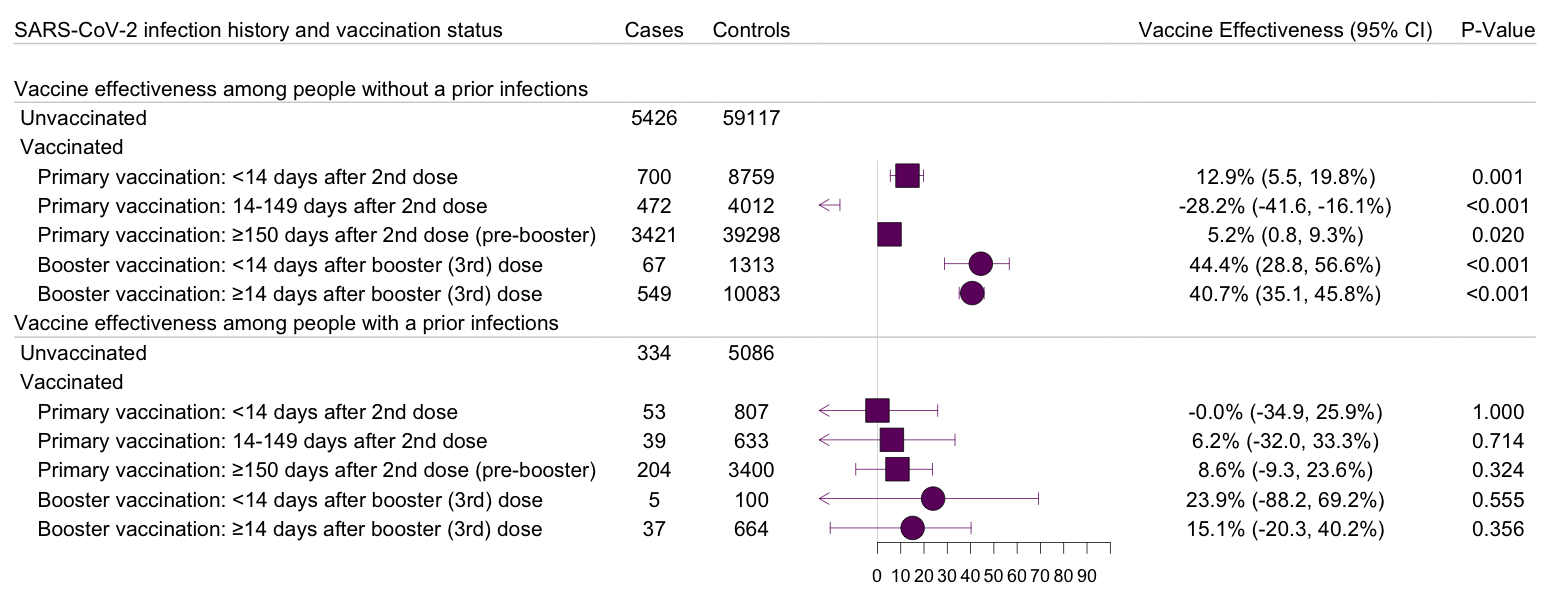


*All Controls:* To limit within person correlation within our controls, we limited the number of control events a person could contribute to three. Here we remove that restriction and allow for the inclusion of all controls.

**Fig G: Forest Plot of Vaccine Effectiveness Including all Controls, Stratified by the History of a Prior SARS-CoV-2 Infection**

A. Adjusted

**
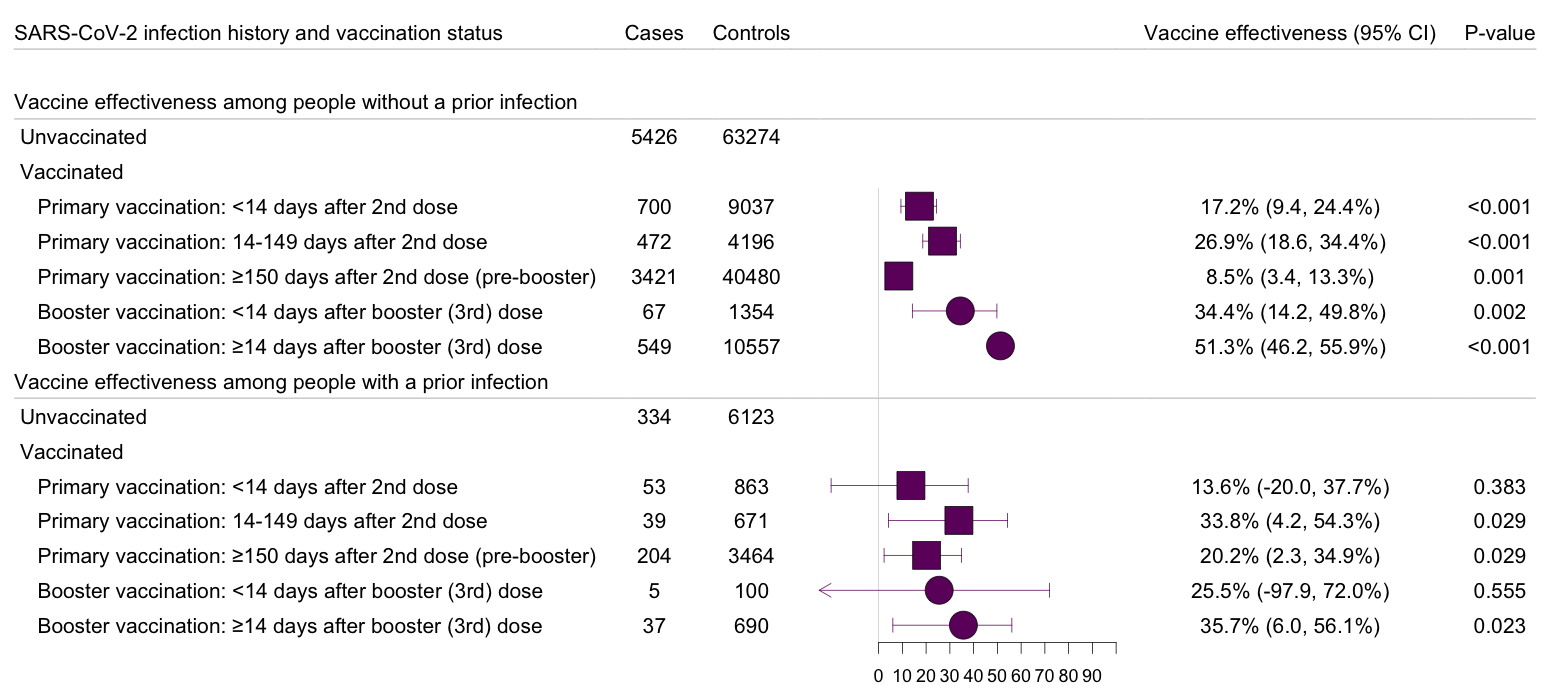
**

B. Unadjusted

**
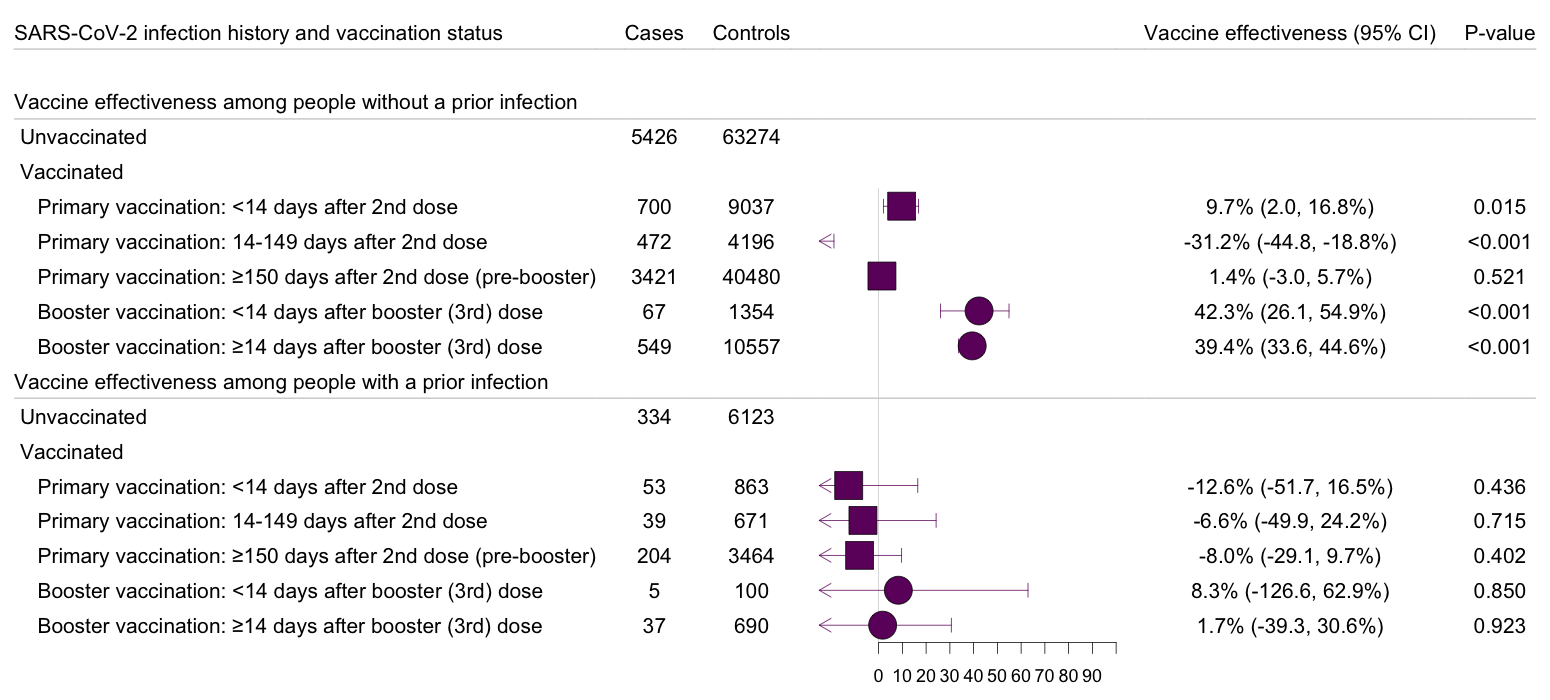
**

*Exclusion of tests collected among people with a prior infection after their first vaccination dose (prior breakthrough infection):* In the primary vaccine effectiveness analysis, we included breakthrough infections, or infections after a vaccination. This allowed us to give estimates of the level of protection vaccinated people with any prior infection have relative to people with only an infection. However, it did not directly evaluate the benefits of vaccination among people who have already been infected with SARS-CoV-2. Here, we excluded tests among people with prior infections that occurred after a vaccine dose.

**Fig H: Forest Plot of Vaccine Effectiveness Excluding Tests Collected Among People with Prior Infections After a Vaccine Dose, Stratified by the History of a Prior SARS-CoV-2 Infection**

A. Adjusted


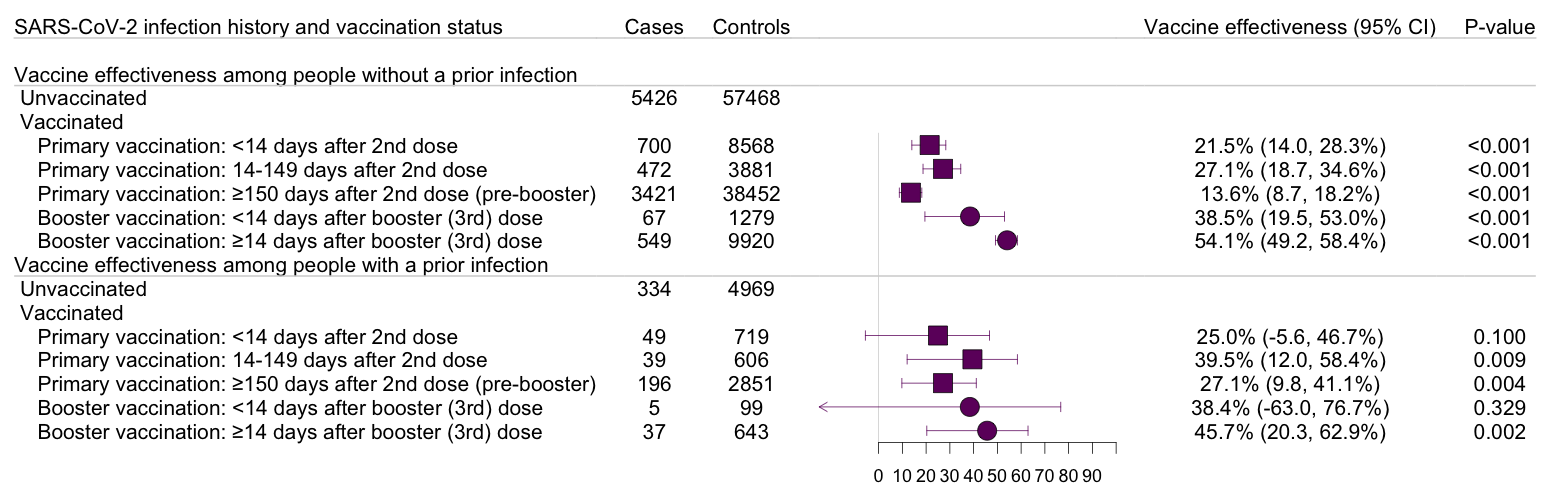


B. Unadjusted

*
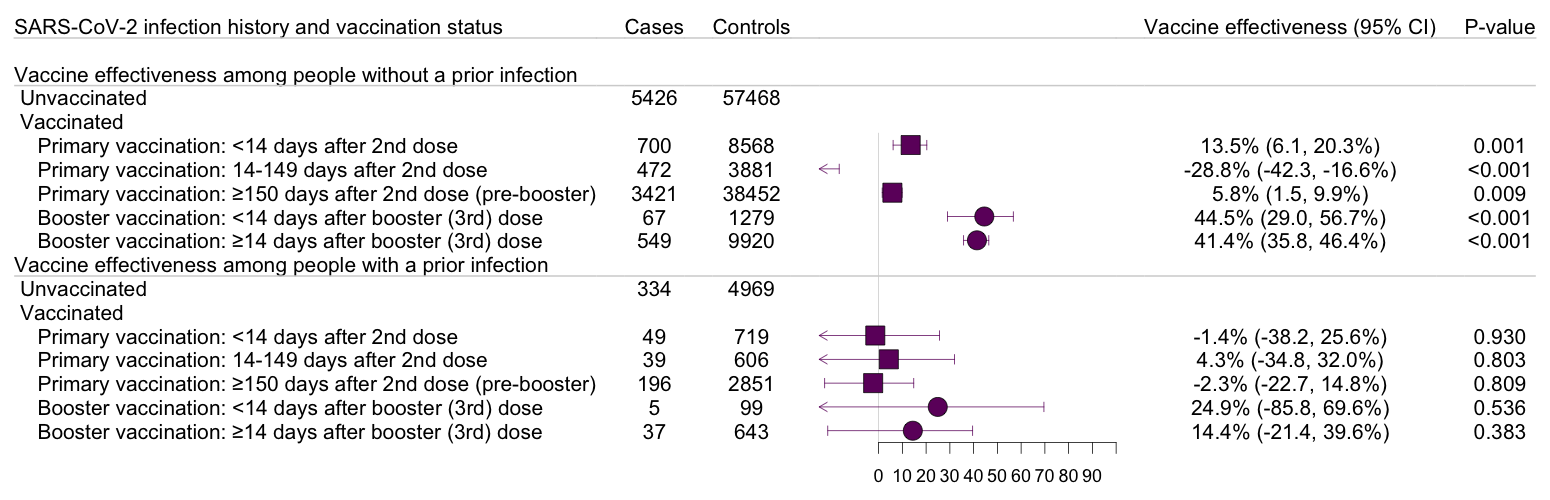
*

*Adjusting for time since referenced vaccine dose:* To examine the impact of booster doses among booster eligible people, we compared the odds of Omicron infection between boosted and booster eligible people stratified by presence of prior infection. In the primary analysis, the estimates came from a single model inclusive of all tests. Because of this, we were limited in our ability to adjust for time since primary series completion. Here, we account for time since primary series completion by restricting our sample to people who received at least two mRNA vaccine doses prior to testing. We, then adjusted for time since first primary series vaccine dose in a continuous manner using a natural spline with 3 knots.

| **Table A. Risk of SARS-CoV-2 Omicron Variant Infection among People Who Received Booster Vaccination Relative to Booster-Eligible People, According to History of a Prior SARS-CoV-2 Infection** | | | | | | |
| --- | --- | --- | --- | --- | --- | --- |
|  |  |  | **Adjusted^b^** | | **Unadjusted** | |
| **Prior SARS-CoV-2 infection history and vaccination status^a^** | **Cases** | **Controls** | **Odds Ratio^c^** | **P-value** | **Odds Ratio^c^** | **P-value** |
| **With a documented prior infection^d^** |  |  |  |  |  |  |
| Booster eligible, ≥150 days after 2^nd^ dose (pre-booster dose)^e^ | 204 | 3342 | - | - | - | - |
| Boosted, ≥14 days after booster (3^rd^) dose^f^ | 37 | 643 | 0.75 (0.50, 1.13) | 0.165 | 0.94 (0.66, 1.35) | 0.748 |
| **Without a documented prior infection^d^** |  |  |  |  |  |  |
| Booster eligible, ≥150 days after 2^nd^ dose (pre-booster dose)^e^ | 3421 | 38446 | - | - | - | - |
| Boosted, ≥14 days after booster (3^rd^) dose^f^ | 549 | 9920 | 0.57 (0.51, 0.63) | <0.001 | 0.62 (0.57, 0.68) | <0.001 |
| ^a^ Due to booster eligibility at the time of the analysis, this analysis was limited to persons 12 years or greater, resulting in the exclusion of 6 booster eligible controls | | | | | | |
| ^b^ Adjusted for date of test, age, sex, race/ethnicity, insurance, Charlson Comorbidity Score, SVI (Social Vulnerability Index) of zip code, municipality, and number of non-emergent visits during the year prior to vaccine rollout in Connecticut (December 2nd 2019 and December 1st 2020) in all analyses and time between testing and last prior infection in analyses of people with prior infection. | | | | | | |
| ^c^ Point estimate (95% confidence interval) | | | | | | |
| ^d^ Documented prior infection defined as a positive RT-PCR or rapid antigen test at least 90 days prior to included test | | | | | | |
| ^e^ Limited to booster eligible people, booster eligible defined as primary series recipients aged 12 years or more who completed their primary series (2-doses) 150+ days prior to the test and were yet to receive a booster (third) dose; 150 days was selected as it reflects the CDC booster recommendations at the time of manuscript submission | | | | | | |
| ^f^ Six tests were collected among people with a prior infection that occurred following their booster dose and were removed from this analysis | | | | | | |
